# The Burramys Project: a conservationist's reach should exceed history's grasp, or what is the fossil record for?

**DOI:** 10.1098/rstb.2019.0221

**Published:** 2019-11-04

**Authors:** Michael Archer, Hayley Bates, Suzanne J. Hand, Trevor Evans, Linda Broome, Bronwyn McAllan, Fritz Geiser, Stephen Jackson, Troy Myers, Anna Gillespie, Chris Palmer, Tahneal Hawke, Alexis M. Horn

**Affiliations:** 1 PANGEA Research Centre, University of New South Wales, Sydney, New South Wales 2052, Australia; 2 Centre for Ecosystem Science, School of Biological, Earth and Environmental Sciences, University of New South Wales, Sydney, New South Wales 2052, Australia; 3 Australian Ecosystems Foundation Inc., 35 Crane Road, Lithgow, New South Wales 2790, Australia; 4 Office of Environment and Heritage, PO Box 733, Queanbeyan, New South Wales 2620, Australia; 5 Physiology, School of Medical Sciences, University of Sydney, Sydney, New South Wales 2006, Australia; 6 Centre for Behavioural and Physiological Ecology, Zoology, University of New England, New South Wales 2351, Australia; 7 Biosecurity NSW, NSW Department of Primary Industries, Orange, New South Wales 2800, Australia; 8 Sanibel-Captiva Conservation Foundation, Sanibel, FL 33957, USA

**Keywords:** *Burramys*, translocation, conservation introduction, fossil, Australia

## Abstract

The fossil record provides important information about changes in species diversity, distribution, habitat and abundance through time. As we understand more about these changes, it becomes possible to envisage a wider range of options for translocations in a world where sustainability of habitats is under increasing threat. The Critically Endangered alpine/subalpine mountain pygmy-possum, *Burramys parvus* (Marsupialia, Burramyidae), is threatened by global heating. Using conventional strategies, there would be no viable pathway for stopping this iconic marsupial from becoming extinct. The fossil record, however, has inspired an innovative strategy for saving this species. This lineage has been represented over 25 Myr by a series of species always inhabiting lowland, wet forest palaeocommunities. These fossil deposits have been found in what is now the Tirari Desert, South Australia (24 Ma), savannah woodlands of the Riversleigh World Heritage Area, Queensland (approx. 24–15 Ma) and savannah grasslands of Hamilton, Victoria (approx. 4 Ma). This palaeoecological record has led to the proposal overviewed here to construct a lowland breeding facility with the goal of monitoring the outcome of introducing this possum back into the pre-Quaternary core habitat for the lineage. If this project succeeds, similar approaches could be considered for other climate-change-threatened Australian species such as the southern corroboree frog (*Pseudophryne corroboree*) and the western swamp tortoise (*Pseudemydura umbrina*).

This article is part of a discussion meeting issue ‘The past is a foreign country: how much can the fossil record actually inform conservation?’

## 1. Introduction

The nineteenth-century poet Robert Browning declared ‘Ah, but a man's reach should exceed his grasp, or what's a heaven for?’ He clearly valued envisioning ideas beyond those within easy reach. Modern conservationists are increasingly prepared to consider innovative strategies that might improve outcomes. Among these is the introduction of species to locations where the species does not currently occur [1,2]. This has been referred to as ‘conservation translocation’ [3] if it is into an area where the species previously occurred or ‘conservation introduction’ if it is into an area where it is not known to have occurred [2].

Most global translocation proposals have been based on distribution data obtained from reliable historical records or predictive models based on the same data. In Australia, translocation programmes have been based on distribution data accumulated since European colonization in 1788 as well as predictive models that have been in turn based on those historical records. For example, the western quoll (Marsupialia, Dasyuridae, *Dasyurus geoffroii*), which is listed by IUCN as Vulnerable, naturally occurs today only in the southwestern corner of Western Australia [4]. However, historical records demonstrate that it was present in all mainland states of Australia when Europeans arrived [5]. Several experimental programmes are underway to translocate it into suitable areas of South Australia [6] and New South Wales [7].

While historical data are arguably the least risky guide for translocation projects, in some cases, wider temporal as well as environmental considerations may be as or even more useful [8]. The Makauwahi Cave Project on the Hawaiian island of Kauai [9] has as a goal, based on excavation of Holocene cave deposits, reconstitution of as much as possible of the local ecosystem that existed before arrival of humans. In Australia, information obtained from some Holocene owl pellet deposits has also been considered relevant in discussions about reconstructing pre-modern species distribution maps [10].

Other proposals based on longer timeframes have made use of Pleistocene distribution data [2,11]. Examples include the trophic rewilding of Pleistocene Park in Siberia [12] as well as the Takahē Project in New Zealand. The takahē (*Porphyrio hochstetteri*), an Endangered alpine bird, was in significant decline. By 1982, only 118 individuals remained [13]. Awareness that it had occupied the lowlands of New Zealand during the Pleistocene led to successful experimental translocation onto lowland islands in Fiordland [14].

Perhaps not surprisingly, some of today's threatened species, like the takahē, are restricted to relatively small, high altitude refuges near the edge of their originally wider range. Other examples of this kind include the giant panda (*Ailuropoda melanoleuca*) and the eastern black-crested gibbon (*Nomascus nasutus*). Distribution contractions of this kind have been concluded, e.g. by Fisher [15], to result from range eclipsing driven by negative factors, human or otherwise, that acted on the core lowland habitat for these species. Having been driven and/or restricted to peripheral, non-optimal higher elevation refuges, they are more likely to become threatened and hence prone to extinction [15]. The translocation of populations back into core lowland habitats, once the negative factors have been removed, might be one of the best ways to optimize the survival prospects of the species, as has proved to be the case for the takahē.

Drawing on palaeoenvironmental evidence, we argue here that clues to potential refugia for threatened species could come from the fossil record much further back in time than the Pleistocene. Here, we focus on the Critically Endangered [16] mountain pygmy-possum (Marsupialia, Burramyidae, *Burramys parvus*; aka ‘MPP’). On the basis of the fossil record, we are advocating its translocation into an environment new for this species but normal for previous species in the same genus. As such, this would constitute a ‘conservation introduction’ [2]. Therefore, the aims of this study were to review: (i) current and developing threats to the survival of this species and (ii) the palaeontological history of its generic lineage to deduce palaeoenvironmental factors to develop an experimental strategy for conserving this Critically Endangered marsupial lineage.

## 2. *Burramys parvus*: critically endangered species caught between a rock and a hot place

**Figure 1.**
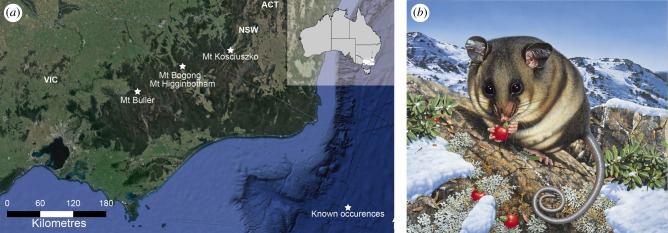
(*a*) Locations of the three populations of mountain pygmy-possums (based on Google map data). (*b*) Adult *B. parvus* with berries from the mountain plum-pine (artwork by Ego Guiotto courtesy of the Australian Geographic Society).

*Burramys parvus* Broom, 1895 (figure 1) was named on the basis of a fossil recovered from a block of cave limestone found near Wombeyan, New South Wales. The precise age of this deposit is unknown but has been reasonably interpreted to be Pleistocene [17]. It was concluded by Broom to represent a previously unknown extinct marsupial that was a phylogenetic link between possums and kangaroos [18–20]. Among its distinctive features are plagiaulacoid upper and lower last premolars. This unusual premolar shape, which results in a vertically fluted/ridged elongate tooth with a cutting edge that resembles that of a circular saw, has occasionally evolved in other mammals such as multituberculates, extinct paucituberculatan marsupials and a few much larger omnivorous kangaroos (e.g. hypsiprymnodontids). Among Australian possums, it is only known to occur in species of *Burramys*.

Sixty-one years after its description, Ride [17] concluded that this species was an unusual kind of possum with no links to kangaroos. His hypothesis was supported when a live individual discovered in 1966 in a ski lodge in Mount Hotham, Victoria, revealed it to be a highly distinctive pygmy-possum [21,22]. The enigmatic fossil had come back to life.

Since 1966, field and laboratory research into the morphology, ecology, diet, reproduction, physiology, genetics, distribution and history of maintenance in captivity of MPPs has occurred [23–26]. They are restricted to alpine/subalpine habitats at or above the snowline in three isolated areas: Kosciuszko National Park in NSW and Mount Bogong-Mount Higginbotham and Mount Buller in Victoria (figure 1) for a total area [27] of less than 7 km^2^. Estimates of population size [25–27] suggest approximately 2000–3000 adults, with numbers rising in wet years and declining in drought years. Population declines can be severe; between 1996 and 2010, there was an estimated 42 and 87% drop in numbers at two of the three populations [28] coincident with the millennium drought [28,29]. Survival at high-elevation sites depends on snow cover in winter to insulate MPPs from freezing air above while hibernating below the surface in cool, humid rock piles [25,27], and an adequate food supply (e.g. bogong moths, *Agrotis infusa*, and seeds in berries of the mountain plum-pine, *Podocarpus lawrencei*) when they emerge from hibernation [30]. Both of these requirements are now under threat.

It is ironic that this ‘Lazarus possum’ is threatened again with extinction. Because there are no higher areas to which it can retreat nor suitable corridors to disperse further south as the climate warms, it appears that under projected climate change scenarios [31] extinction of this high-elevation specialist species in the wild is inevitable. This is why the IUCN has classified this species as Critically Endangered despite the estimated population size exceeding 2000 individuals [27]. Although the MPP recovery plan [28] recommends ways to reduce immediate risks, there is now no way to avoid climate heating and drying in the alpine areas. The plan does, however, acknowledge the importance of ongoing investigations, viz this project, into whether MPPs could persist outside the alpine zone.

## 3. Threats to the survival of the mountain pygmy-possum

Mountain pygmy-possums are sensitive to fluctuations in environmental temperatures: individuals that are unable to find adequate shelter—such as they find naturally in rock piles in the alpine zone—are known to die at temperatures above 28°C [32]. Global heating may push alpine temperatures upto and above this threshold [31,33,34], in which case any lack of suitable thermally buffered refugia will expose these pygmy-possums to this new thermal stress. Beyond this longer-term issue, immediate threats include habitat loss, degradation and fragmentation from ski-resort development [26,35]. Approximately 40% of NSW and 80% of Victorian MPPs occur in habitat adjacent to or within ski-resort lease areas [26]. Currently, the MPP is ranked as one of the Australian species most vulnerable to the impacts of climate change [28,31,33,34,36]. Another species, the Bramble Cay melomys (*Melomys rubicola*) was last seen in 2009 and now appears to be extinct [37,38] owing to ocean inundation from rising sea levels caused by climate change. In the case of MPPs, climate heating and habitat loss compound other threats that include predation from cats and foxes [23,31], weed invasion [28], availability of seasonal and migratory food items like bogong moths (*Agrotis infusa*) [39,40] and exposure to disturbances while hibernating [24,41]. Hibernacula are normally maintained at between 1.5 and 2.5°C [41]. If they fall lower than 0.6°C, which can result from the loss of snow insulation, MPPs will arouse frequently from torpor to maintain core body temperature by shivering, which increases energetic costs and the threat of starvation during winter [24,41]. Of immediate current concern is the catastrophic decline in bogong moths in the alpine zone [39]. These fat-rich insects are a resource required by these possums as soon as they arouse from hibernation [27,31,40].

## 4. Background to the plan to conserve this lineage of Critically Endangered possums

**Figure 2.**
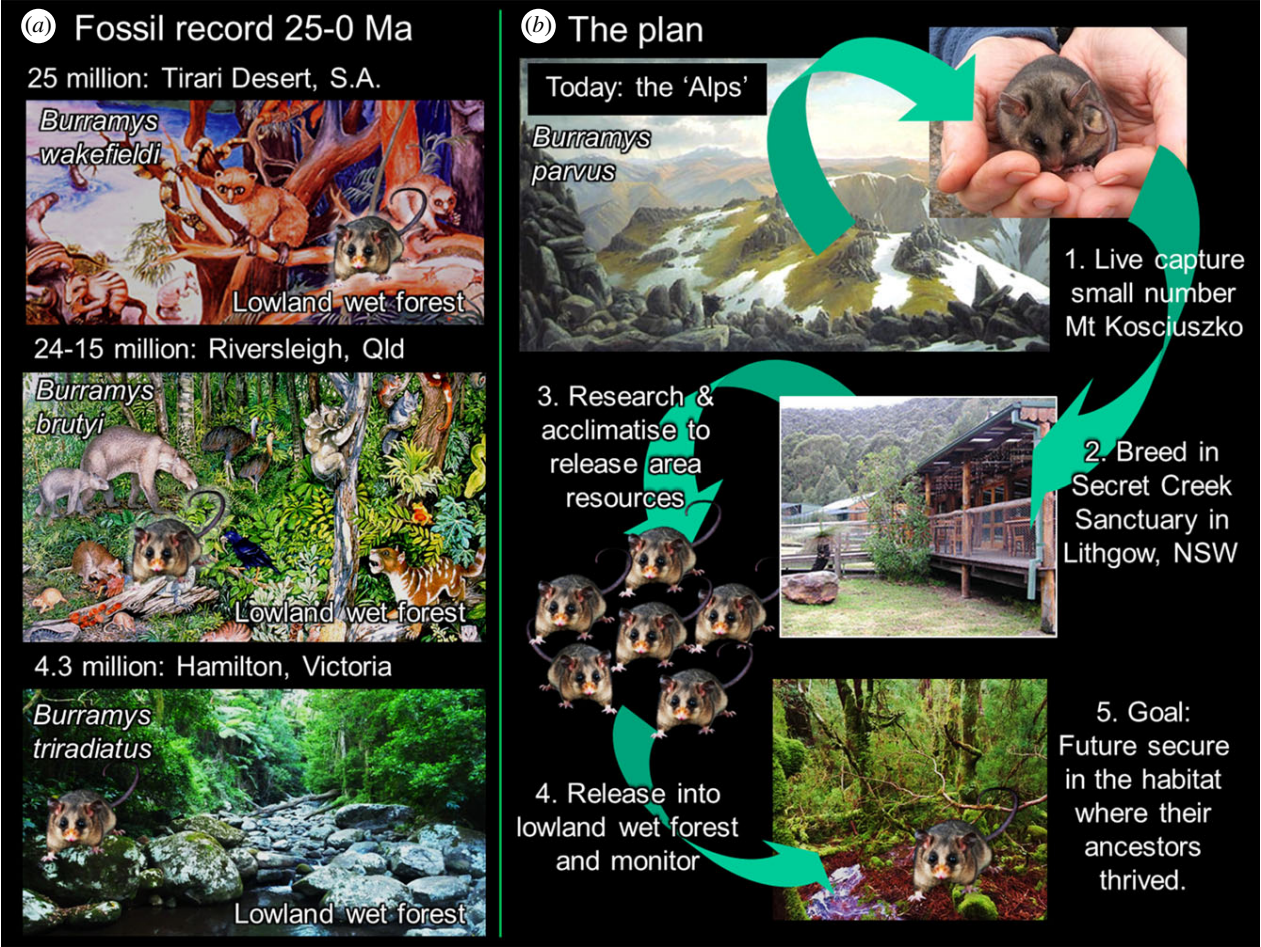
(*a*) Earlier species of *Burramys* are all known from fossil deposits accumulated in cool, temperate, lowland wet forest environments. Reconstruction of Late Oligocene habitat of *B. wakefieldi* in northern South Australia (J. Reece). Reconstruction of Miocene habitat of *B. brutyi* in the Riversleigh region of northwestern Queensland (D. Dunphy). Modern wet forest similar to Early Pliocene habitat of *B. triradiatus* in NW Victoria (M. Archer). (*b*) The conservation introduction proposal outlined in this paper. Painting of Mt Kosciuszko region (Von Guerard 1863). *Burramys parvus* in hands (H. Bates). Secret Creek facilities (M. Archer). Rainforest release site (M. Archer). Replicated small images representing *Burramys* possums are based on a photograph by J. Sartore.

As palaeontologists documenting the prehistory of species of *Burramys*, we have suggested since 1991 [31,42,43] that there is a novel potential strategy to conserve MPPs. The strategy is based on the fossil record, which indicates that all pre-Quaternary species of *Burramys* (*B. wakefieldi*, *B. brutyi*, *B. triradiatus*) thrived in lowland, wet forest palaeocommunities (figure 2). Pleistocene populations of MPPs may also have occupied lowland wet forests, but the current fossil record is inadequate to test this possibility.

*Burramys wakefieldi*, from the species-diverse Ngama Local Fauna, Lake Palankarinna, Tirari Desert, northern South Australia [44], occurred in lowland, wet forests that surrounded lakes and rivers [45]. The age of the assemblage has been interpreted to be 24.7 and 25.0 Ma [46].

**Figure 3.**
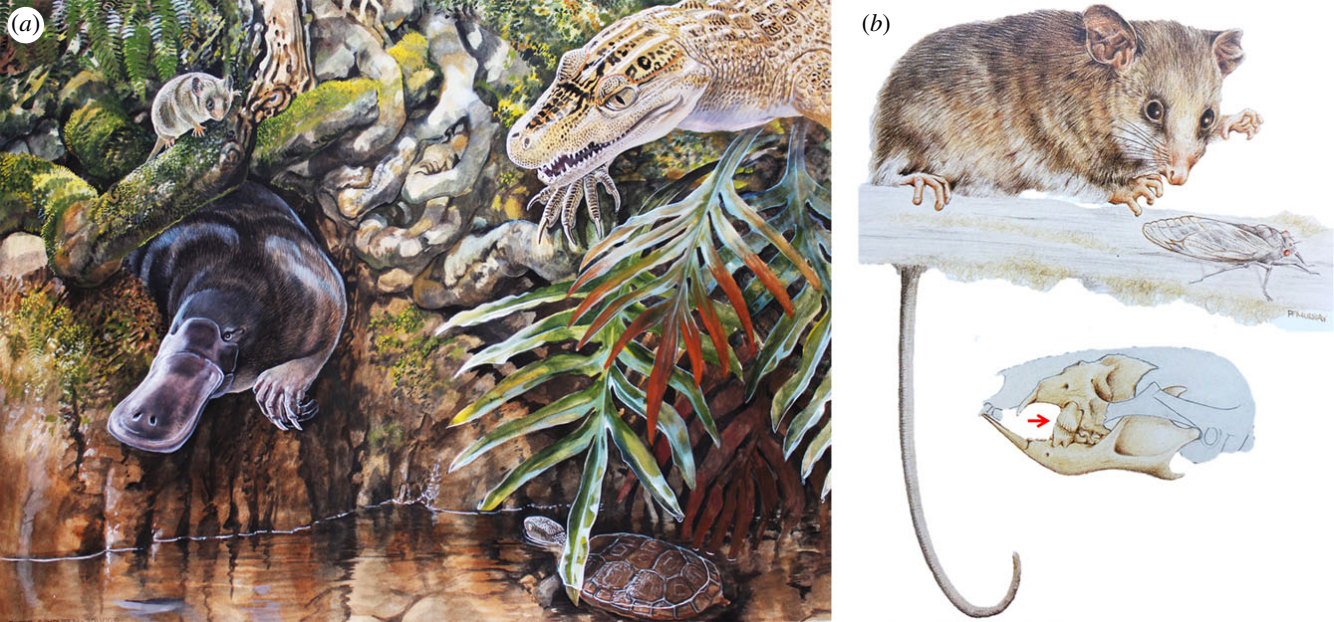
(*a*) Reconstruction of a lowland closed forest palaeoenvironment based on Ringtail Site, Riversleigh. Animals in this Middle Miocene faunal assemblage include *B. brutyi*, the toothed ornithorhynchid platypus *Obdurodon dicksoni*, the mekosuchine terrestrial crocodile *Trilophosuchus rackhami* and a species of the chelid turtle genus *Pseudemydura* similar to if not conspecific with the living Critically Endangered *P. umbrina*. (Artwork by P. Schouten.) (*b*) Like all species of this genus, *B. brutyi* had highly distinctive plagiaulacoid posterior premolars (indicated by the arrow). (Artwork by P. Murray [47].)

*Burramys brutyi* (figure 3) is abundant in most Late Oligocene (25 Ma) to at least late Middle Miocene (approx. 13 Ma) species-diverse faunal assemblages from the Riversleigh World Heritage Area in NW Queensland [47–51]. It is also possible that it persisted until early Late Miocene time although the relevant local fauna at Riversleigh, the Encore Local Fauna (LF), has not yet been radiometrically dated. Riversleigh's Late Oligocene deposits may be slightly younger than the Late Oligocene deposits in central Australia [46,52]. Ages of Riversleigh's Early, Middle and possibly Late Miocene deposits are based on radiometric dating as well as (in the case of the probably Late Miocene Encore Site) biocorrelation [53]. While vegetation and climate of the Late Oligocene of this area are uncertain, it is clear that in at least the Early and Middle Miocene, this area supported cool, temperate, well-watered, lush closed forest [47,50]. Significant differences in fossil frog assemblages in the Encore Local Fauna suggest a reduction in rainfall had begun to occur by early Late Miocene time [45].

*Burramys triradiatus* occurs in the species-diverse Early Pliocene Hamilton Local Fauna of southwestern Victoria [54]. The palaeohabitat was humid, wet, lowland forest [55]. A basalt that overran the fossil soil that contained the bones has been radiometrically dated at 4.35 Ma [56].

*Burramys parvus* occupies restricted, species-depauperate rocky habitats in alpine/subalpine regions of New South Wales and Victoria at elevations ranging from approximately 1200 to 2228 m.a.s.l. [57]. Its dependence on rock piles in these habitats relates to this species' need to be insulated (buffered) from the extended alpine temperature range found in both winter and summer [58] as well as the evident need to be near water that flows from underground springs in the occupied rock piles [26]. Humid microenvironments appear to have been a critical requirement or at least correlate for all species of *Burramys*.

MPPs have been reported from Pleistocene cave deposits in areas of New South Wales and Victoria [56], such as Wombeyan (556 m.a.s.l.), Buchan (121 m.a.s.l.) and Jenolan Caves (e.g. Nettle Cave; 839 m.a.s.l.). The precise times of occupation of these topographically relatively lower areas are less clear, but it is possible that they correlated with colder intervals of the Pleistocene when the snowline extended further downslope than it does today [59].

Based on the faunal assemblages in these Pleistocene deposits, it is clear that these communities were far more speciose than those that contain living MPPs. For example, in Nettle Cave, MPPs coexisted with at least 23 other kinds of terrestrial mammals [59] in contrast with only six that coexist with MPPs today [26]. These Pleistocene taxa, all still-living species, include arboreal forms such as sugar gliders (*Petaurus breviceps*) and brushtail phascogales (*Phascogale tapoatafa*), which suggests the presence of forests or at least woodlands in the area adjacent to Nettle Cave.

The fact that all known faunal assemblages from the Late Oligocene to Pleistocene that contain species of *Burramys* were highly species-diverse, in distinct contrast to those that contain MPPs in the alpine zone today, suggests that the modern depauperate situation is aberrant and probably a reflection of the extreme conditions that characterize the alpine/subalpine zone. Hence, the introduction of MPPs into contemporary, lowland, more mesic species-diverse communities would be re-establishing the geologically/palaeontologically more ‘normal’ community structure for species of this genus.

Pliocene to Quaternary pollen records [60–63] suggest that different forest types have been expanding into and out of upland areas of the Great Dividing Range in cycles driven by climatic fluctuations. At least 50 such climatic episodes have been documented in Australia [63]. It seems probable that ancestral lowland populations of MPPs, along with other plant and animal taxa that occupied the same lowland wet forest habitats, expanded into alpine zones during warm, wet climate intervals. As the climate cooled and snow fell annually, MPPs evidently came to depend on cool, humid rock piles for hibernation as well as avoidance of high summer temperatures. Simultaneous loss of continuous areas of wet forest vegetation on mountain flanks would have, as it does now, prohibited these possums from dispersing downslope to climatically less stressful forests of the lowlands. The three geographically isolated alpine/subalpine populations of MPPs are now genetically distinct from one another and have been interpreted [64] to have had disjunct populations since the mid-Pleistocene (420–680 ka).

Bates [26] documented a range of situations in which MPPs have been maintained in warmer ambient lowland conditions, including Healesville Sanctuary (Victoria), Taronga Zoo (NSW), Canberra (ACT) and Jindabyne (NSW). Although they did not hibernate, they did enter torpor during the relatively warmer winters of Canberra. In Sydney and Canberra, they exhibited aseasonal breeding. While ambient temperature ranges in these earlier colonies were not recorded, either summer temperatures in the colonies were below 28°C or the MPPs involved were provided with cooler areas to which they could retreat. Natural retreats such as rock piles or other insulating materials would provide sufficient shelter of a similar kind in any lowland forests that were selected as potential release sites for captive-bred MPPs, such as Secret Creek Sanctuary in Lithgow.

Geiser [65] demonstrated that another burramyid, the eastern pygmy-possum (*Cercartetus nanus*), a species that uses bouts of torpor in the wild to survive short periods of hardship, had the capacity in the laboratory to hibernate for 367 days, using a fraction of the energy needed when normothermic. Clearly, the ability of at least some burramyids to adapt their physiological responses to different conditions is greater than contemporary ecological studies alone would suggest. However, the potential ability of MPPs to adapt to otherwise lethal conditions in the alpine/subalpine zone triggered by climate change are limited by two thresholds: exposure to lethal temperatures following the loss of snow cover in winter, which could happen relatively suddenly at any time [31]; and lack of an essential source of food of the kind and volume currently provided by bogong moths, which are rapidly disappearing [39] as climate change progresses.

Considering the evolutionary record as a whole, there is no evidence that there was ever more than a single species of *Burramys* alive at any one time. Each of the four species, all of which are small (less than approx. 50 g), is distinguished from the others by relatively minor dental features such as the specific number of ridges on the flank of the plagiaulacoid premolars. We interpret this to suggest that these four species, which form a chronocline of essentially very similar species spanning 25 Myr, may represent an anagenetic lineage whose members have occupied the same basic ecological niche in wet forests in lowland areas over this period of time. Although it is possible that some as yet unknown pre-modern species of *Burramys* occupied a different niche in highland or lowland areas, there is no evidence that this was the case. Hence, we suggest the high probability that the ancestral *B. parvus* population was similarly adapted to wet forest conditions and expanded with these forests up into the subalpine/alpine zone during one of the interstadial periods of the Pleistocene where, with a return of stadial conditions, it was forced to adapt to conditions that were suboptimal for the lineage as a whole.

Our hypothesis then, based on this background understanding, is that this lineage of dentally highly distinct species of *Burramys* has been from at least the Late Oligocene adapted to thrive in cool, temperate, low-elevation, wet forests. While we do not know when or under what conditions its most recent member, the MPP, became isolated in the subalpine/alpine zone, like its predecessors, it retains the same distinctive dental specialisations, small body size, requirement for high humidity [26] and lack of exposure to extreme temperatures. Because no other small mammal, let alone any other possum, in Australia's modern forests has remotely similar dental specializations, we suggest that if MPPs were to be introduced into lowland wet forest environments, there is little or no likelihood that they would compete with other mammals in those habitats.

## 5. Planned steps to implement the palaeo-based experiment

Our plan (figure 2*b*) to use the fossil record to develop a strategy for conserving the *Burramys* lineage was anticipated in 1991 [43] and broadly discussed in 2012 [31]. Here, we present 16 steps (table 1) to facilitate this plan. Planning and relocation recommendations by IUCN/SSC [3] and others [70–72] have been taken into consideration. With regard to temperatures at or above 28°C, we have recorded 2 years of temperature records (2017/2018) at Secret Creek Sanctuary in Lithgow, New South Wales, one of the potential release sites. Temperatures were continuously measured in above-ground rock piles anticipated to be shelter retreats within a breeding facility anticipated for this species (table 1). Over the 2-year period, winter temperatures averaged −4°C while summer temperatures never exceeded 24°C. Therefore, given access to shelter within areas such as this, ambient temperatures in low-elevation forested areas would not become a limiting factor.

**Table 1.** Sixteen challenges for the palaeo-based conservation introduction experiment.

step	description	progress to date (2019)
1	multi-disciplinary team of ecologists, mammalogists, palaeontologists, palaeoecologists, reproductive biologists, ecophysiologists and husbandry specialists	*done* (authors of this paper and [31])
2	document morphological, environmental, palaeocommunity changes over time	*done* [26,47,49]
3	investigate environmental threats to and survival requirements of living MPPs	*done* [23,25,26,31,57,66]
4	determine ecophysiological resilience to assess potential capacity of MPPs to adapt to non-contemporary situations	*done* [24,26,41,65,67]
5	document and assess results of early efforts to maintain colonies in lowland sanctuaries and zoos	*done* [26]
6	collaborate with an established sanctuary where a non-alpine breeding facility for MPPs can be built	*done* (Secret Creek Sanctuary, Lithgow, NSW; elevation a.s.l. 1000 m; permanently shaded area where temperatures range from −4°C in winter to 24°C in summer)
7	raise funds to help construct the breeding facility in Secret Creek Sanctuary	*ongoing* ($150 000 already raised by National Parks Association, Australian Geographic Society & UNSW)
8	focus academic and public attention on this research programme as a flagship project about strategies to address climate change threats	*done* ([68]; also formal part of UNSW's ‘Climate Change Grand Challenge’ initiative)
9	ensure compatibility with recommendations of commonwealth government ‘National Recovery Plan’	*done* (meets *Objective 5* of plan)
10	collaborate with regulatory agencies to obtain and manage a captive colony	*ongoing* (with tacit approval to proceed)
11	conduct behavioural and other research on the captive colony to assess resilience as well as acclimatize MPPs to new foods	*anticipated* (research students and staff will conduct experiments prior to trial releases)
12	acclimate mothers and pouch/nest young to warm conditions to try to generate, via phenotypic plasticity, individuals better able to cope with high temperatures	*anticipated*
13	assess genetics of individuals to be released	*anticipated*
14	identify optimal locations for trial releases	*anticipated*
15	monitor introductions for at least two decades to assess outcomes of experimental release	*anticipated*
16	use results as a basis for considering introductions of other alpine species that lack fossil records	*anticipated* (e.g. potential candidate is critically endangered corroboree frog [69])

## 6. Considering and managing risks: Q&As

*Could introduction of MPPs into lowland wet forest communities lead to negative impacts on those communities*? We hypothesize that the introduction will result in reoccupation of an otherwise vacant niche that, prior to the Pleistocene, was normally and only filled by species of *Burramys*. Hence, we predict there will be no concomitant declines in other endemic species. Preliminary analyses of biodiversity in Riversleigh's fossil assemblages suggest that the presence of *B. brutyi* correlates with high species diversity, suggesting that they were compatible components of those species-rich communities.

*Will MPPs survive predation in those communities*? Earlier Cenozoic species of *Burramys* thrived in lowland wet forest communities despite a wide range of sympatric reptilian, mammalian and avian predators. Today, the MPP is surviving, albeit with some difficulty, even introduced carnivores such as foxes and cats. Depending on the results of monitoring experimentally released MPPs, programmes to control feral carnivores, such as those now in use in the alpine zone, may be necessary in specific lowland wet forests. Predator-proof fencing may be required until the releases develop appropriate fear/avoidance responses. Before wild release, MPPs could also undergo training to enhance fear of carnivores, a strategy that appears to be at least partially successful in relation to translocation of other marsupials [73].

*Will MPPs be able to adapt to lowland wet forests in terms of climatic conditions*? Myers *et al*. [51] demonstrated a significant change in Riversleigh's Early to Middle Miocene communities between 18.0 and 13.5 Ma, an interval of time when atmospheric gas composition underwent radical change all around the world. *Burramys brutyi* remained an important component in all communities before and after this event despite major changes in overall marsupial diversity, an indication of its apparent environmental resilience to climate changes in lowland areas. Furthermore, it has been determined [26] that captive colonies of MPPs have previously survived in ambient lowland climatic conditions in Victoria and New South Wales, evidently without hibernating. We know that small mammals that use torpor or hibernate are less likely to become extinct [67], thus the physiological flexibility of MPPs should increase their prospects of adapting to the new ambient lowland climatic conditions. Secret Creek Sanctuary itself, where the breeding facility will be established, is densely forested, at 1000 m.a.s.l., receives frosts and some snow, has a rocky substrate with significant thermally insulated refugia and is enclosed by a predator-proof fence, making it one of the potential sites for initial experimental releases.

*Will phenotypic plasticity help MPPs to cope better with high temperatures when raised under warm conditions?* There is plenty of evidence based on placental mammals [74] and some based̀ on marsupials [66] that raising individuals under warm conditions results in a ‘warm phenotype’ without the need for long-term selection. Individuals raised in warm conditions generally have longer appendages than those raised in the cold, show greater heat loss and a reduced expression of torpor. Although we do not know that this resilience is present in the *Burramys* lineage, given its 25 Myr-long history of thriving in relatively warm climates, it may well be a conserved capacity of the genome.

*Will MPPs be able to survive without their customary alpine foods and adapt to resources in their new habitats*? Hawke *et al*. [57] and Schulz *et al.* [75] demonstrated that a lower-elevation population on Mount Kosciusko exhibits different food preferences from those of upland populations, suggesting dietary flexibility in this species. Furthermore, captive colonies have been known to thrive on foods not available in the alpine zone [26]. To further promote successful transition, we plan to acclimatize individuals to foods they will encounter before they are released.

## 7. Discussion

Globally, among continents, Australia has suffered the greatest loss of endemic mammal species with 29 endemic mammals having gone extinct and at least as many more struggling to survive [76] since European colonization in 1788. This represents about one-third of all mammal extinctions in the world over the past 500 years [76]. Faced with the challenge to devise strategies to slow this cascade of extinctions, there is increasing recognition of the potential relevance of the fossil record [77,78].

As noted above, the four species of *Burramys*, which differ from each other in only minor morphological details, form a chronocline spanning the past 25 Myr. Each of the pre-Pleistocene taxa appears to have filled the same currently vacant niche in lowland wet forest communities and to have been part of complex, biodiverse ecosystems. For these reasons, we hypothesize that introducing MPPs into contemporary lowland wet forest communities, where this same niche is vacant, would be unlikely to lead to competition with resident species and would help to conserve this vulnerable highly distinctive lineage of mammals.

While the current population of *B. parvus* is estimated to be about 2500 individuals (L. Broome & D. Heinze 2019, unpublished data), it could crash with just two consecutive years of diminished snowfalls [31] or with continuing drought-induced population declines of bogong moths. Delays in trialling conservation introductions of the kind proposed here involve a significant risk of the kind that led to extinction [5] of the toolache wallaby (*Macropus greyi*). Efforts to translocate this species to secure habitat in 1923 and 1924 were unsuccessful at least in part because by the time the decision to do this was made, there were fewer than 20 individuals left [79].

However, despite expectations, it is conceivable that the subalpine/alpine population of MPPs might be able to survive the climate change that is coming. Because of this possibility, it could be argued that our proposal is a waste of time. We have given reasons to explain why survival of MPPs in the subalpine/alpine zone in the long term is improbable and for these reasons, we suggest that not carrying out this experiment as a ‘safety net’ strategy would be gambling the future of the entire lineage on this one improbable outcome. Further, given that we are proposing to translocate less than 1% of the existing population, 99% of the population will be left in the subalpine/alpine zone to face their albeit low chances for long-term survival. Hence, the impact of this proposed translocation can either be beneficial or neutral but cannot have a negative impact on the subalpine/alpine population.

Our plan has potential outcomes beyond its focus on conserving MPPs. We suggest that if MPPs can be successfully established in lowland wet forest habitats, this could provide incentive to try similar translocations for other sympatric species threatened in the same alpine habitats, such as the Critically Endangered corroboree frog (*Pseudophryne corroboree*) [69,78]. In contrast with MPPs, there are no pre-modern fossil records for the corroboree frog and hence no direct evidence for potentially lower-elevation populations. It might, however, be worthwhile to trial release this frog into any lowland habitat that proves to be an effective sanctuary for MPPs, albeit after consideration is given to potential new risks such as chytrid fungus.

If the MPP conservation introduction we propose here is successful, it could also encourage comparable initiatives focused on endangered lineages of non-alpine taxa. The Critically Endangered western swamp tortoise (*Pseudemydura umbrina*) struggles to survive in Western Australia as increasingly severe climate change-exacerbated droughts threaten its swampland habitat [80]. There are no other known occurrences of this species or genus, living or fossil, with one exception: a Middle Miocene fossil turtle (*Pseudemydura* sp. cf. *P. umbrina*) from a fossil deposit that accumulated in what was a lowland lake in a wet forest at Riversleigh in northwestern Queensland [81]. This record suggests the potential value of trialling a conservation introduction of a founding colony into a cool, temperate forest pool in a wet forest environment.

As extinctions around the world accumulate in response to global heating, we urge that routine consultation should occur with palaeontologists and ecophysiologists who may well have insights that could broaden the range of potential conservation strategies and increase optimism about the future [77,82]. We further suggest that in some cases, the depth of time (the palaeontological ‘reach’) that may be of value for developing conservation strategies could, as we have argued here, extend back in time well prior to the Quaternary.

## Data accessibility

This article has no additional data.
